# Case Report: A case of neonatal osteogenesis imperfecta: navigating critical care and early fracture management through multidisciplinary collaboration

**DOI:** 10.3389/fped.2025.1737710

**Published:** 2025-12-17

**Authors:** Haoqiang Xie, Fuzhen He, Fengmin Wu, Weisen Xu, Ning Li, Xiaoguang He

**Affiliations:** Department of Neonatology, Dongguan Children’s Hospital Affiliated to Guangdong Medical University, Dongguan, China

**Keywords:** osteogenesis imperfecta, neonatal critical care, pediatric trauma, multidisciplinary team, COL1A2 gene, fracture healing

## Abstract

**Background:**

Osteogenesis Imperfecta (OI) poses a unique challenge in pediatric trauma and critical care, where the fragility of bone intersects with life-threatening systemic complications, such as neonatal respiratory failure. The early postnatal period is particularly precarious, demanding a delicate balance between life support and fracture prevention.

**Case presentation:**

A 9-day-old male neonate with prenatally diagnosed COL1A2 (c.1459G > A, p.Gly487Arg) mutation was admitted to our NICU for respiratory distress and pneumonia. He was the progeny of a father with OI, delivered via cesarean section at 34 weeks due to fetal skeletal deformities.

**Management and outcomes:**

A proactive, multidisciplinary team (MDT) protocol was immediately implemented, focusing on non-invasive respiratory support, meticulous handling to prevent iatrogenic injury, and optimized nutrition. This approach successfully resolved his respiratory failure without any new fractures during the NICU stay. However, on the 16th day post-discharge, the infant sustained a spontaneous fracture of the right proximal femur. This was managed conservatively with a Pavlik harness. Follow-up revealed rapid callus formation by day 52 and complete union by day 136, showcasing the characteristic hyperplastic healing pattern of OI.

**Conclusion:**

This case underscores that a coordinated MDT approach is vital for stabilizing critically ill neonates with OI. The occurrence of a fracture shortly after discharge highlights the transition to home care as a period of extreme vulnerability. Empowering families with comprehensive education and ensuring continuity of care are as crucial as in-hospital management for improving long-term outcomes in these fragile infants.

## 1 Introduction

Neonates with severe Osteogenesis Imperfecta (OI), notably Sillence type III, present a complex clinical scenario where profound bone fragility—representing the “trauma” component—coexists with life-threatening systemic compromise, most critically respiratory failure, which defines the “critical care” imperative (1). Managing these infants in the Neonatal Intensive Care Unit (NICU) necessitates a carefully balanced strategy: delivering aggressive respiratory and systemic support while enforcing stringent precautions to prevent iatrogenic injury. This interplay establishes OI as a paradigm condition for multidisciplinary management in pediatric trauma and critical care (2). We report a case of a neonate with a COL1A2 mutation, detailing the multidisciplinary approach from critical respiratory support to the management of a post-discharge spontaneous fracture. This report aims to contribute to the evolving clinical framework for managing these vulnerable infants.

## 2 Case presentation

### 2.1 History

**Figure 1:**
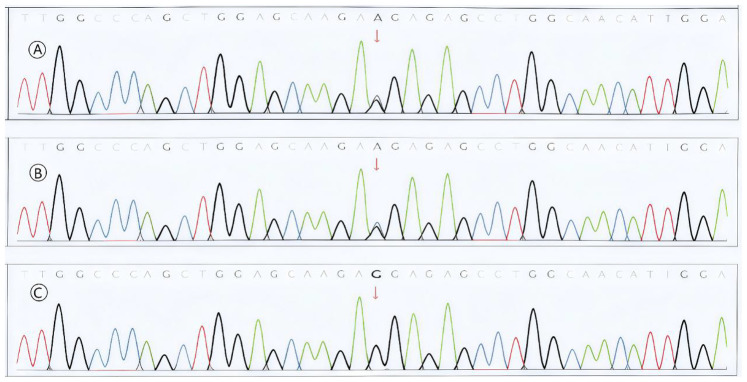
Prenatal genetic diagnosis and familial segregation. **(A)** Whole-exome sequencing (WES) of amniotic fluid cells from the proband, revealing a heterozygous c.1459G > A mutation in the COL1A2 gene (red arrow). **(B,C)** Sanger sequencing validation within the family, confirming the mutation was inherited from the affected father **(B)**, while the mother was wild-type **(C)** This figure establishes the genetic etiology of the disease, providing the definitive basis for prenatal diagnosis and postnatal precision management.

A 9-day-old male neonate was transferred to our NICU in January 2025 for persistent respiratory distress. The infant was born at 34 weeks' gestation via cesarean section due to prenatal ultrasound findings suggestive of OI (bilateral femoral bowing and breech presentation). He was the second child of a father with a confirmed history of OI. Prenatal genetic testing via amniocentesis and whole-exome sequencing identified a heterozygous c.1459G > A (p.Gly487Arg) mutation in the COL1A2 gene, which was inherited from the father (Figure 1).

### 2.2 Physical examination on admission

Examination in the NICU revealed a neonate exhibiting tachypnea (56 breaths per minute), significant tracheal retractions, thoracoabdominal asynchrony, coarse breath sounds with rales bilaterally, and oxygen saturation declining to 85%, necessitating nasal Continuous Positive Airway Pressure (nCPAP) (Figure 4). Notably, bilateral knee valgus deformities and bowing of the lower limbs were observed, consistent with the prenatal diagnosis. The sclerae were not blue.

### 2.3 Investigations

**Figure 2:**
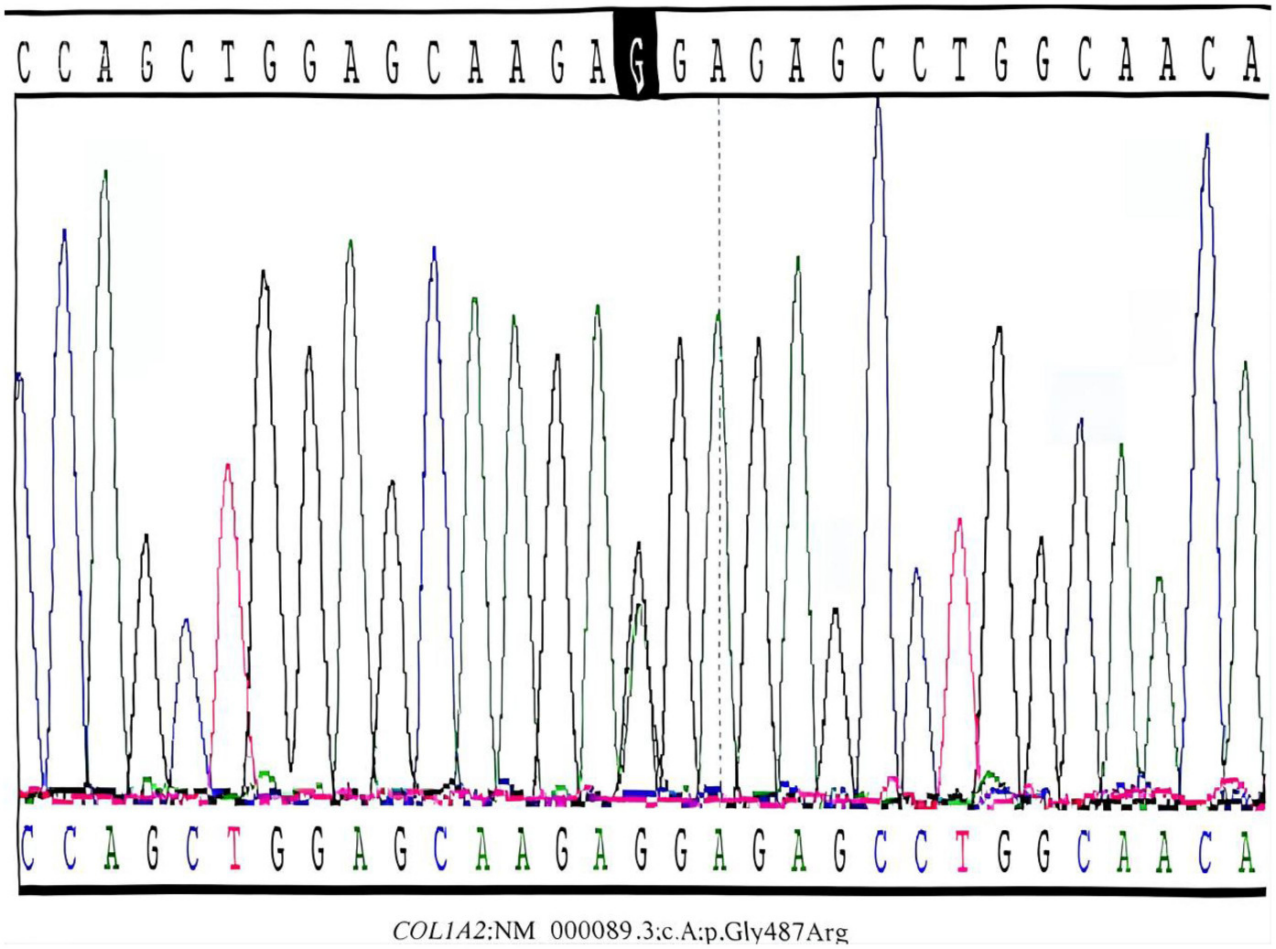
Sanger sequencing confirmation of the COL1A2 mutation in the proband. Postnatal Sanger sequencing validation confirmed the presence of the heterozygous c.1459G > A mutation (red arrow) in the proband's peripheral blood DNA. This missense mutation leads to the substitution of glycine by arginine at position 487 (p.Gly487Arg) within the highly conserved triple-helical domain of the collagen *α*2**(I)** chain. Such structural mutations are typically associated with a severe type III OI phenotype.

**Figure 3:**
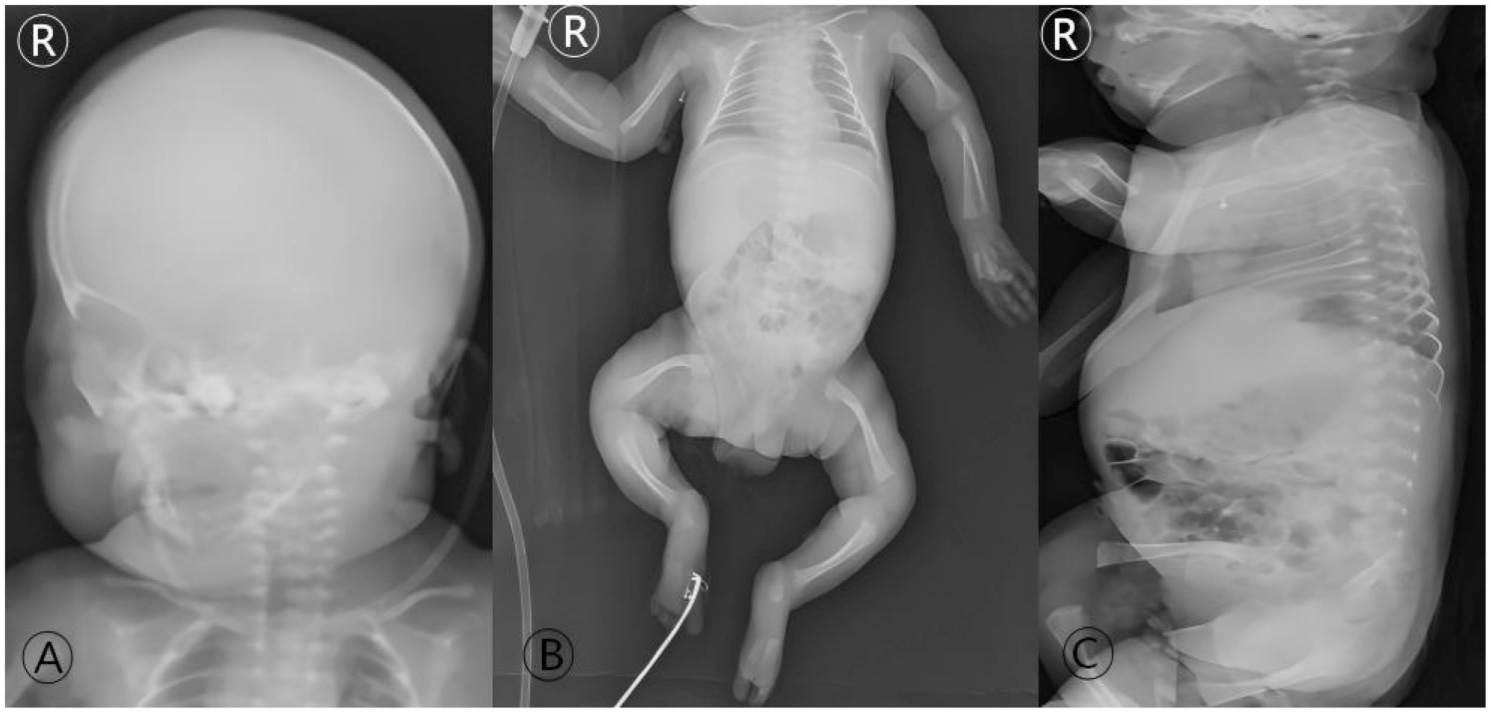
Initial radiographic skeletal survey at admission (Day 9 of life). Full-body radiographs demonstrate the classic imaging features of severe, type III OI: **(A)** diffuse osteopenia and thinning of the cranial vault. **(B)** Generalized osteopenia of the long bones, thin cortices, relatively widened medullary cavities, and flared metaphyses; slender and curved bilateral clavicles are also evident. **(C)** Loss of normal spinal curvatures. These findings form the radiographic basis for the diagnosis of OI in the neonatal period and explain the inherent high fracture risk.

Laboratory findings were significant for elevated interleukin-6 (44.62 pg/mL), suggesting infection/inflammation, while calcium, phosphate, and ALP levels were within normal limits for gestational age, helping to rule out metabolic bone disease (3). Genetic analysis by Sanger sequencing confirmed the COL1A2 c.1459G > A mutation (Figure 2). Skeletal survey radiographs demonstrated generalized osteopenia, thin cortical bones, and bowing of long bones and clavicles, classic for severe OI (Figure 3) (1).

### 2.4 Clinical course, multidisciplinary management, and outcome

#### 2.4.1 NICU phase: multidisciplinary stabilization and prevention

**Figure 4:**
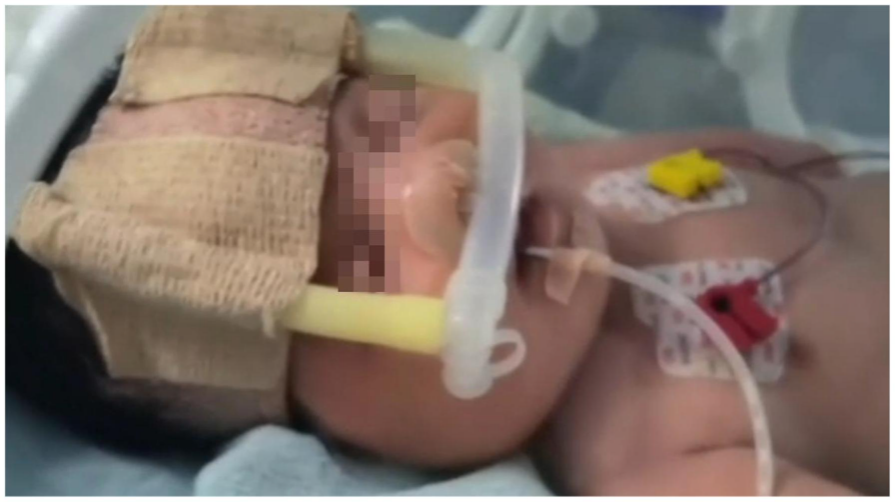
The infant received nasal Continuous Positive Airway Pressure (nCPAP) with the following parameters: positive end-expiratory pressure (PEEP) 5 cmH_2_O, fraction of inspired oxygen (FiO_2_) 30%.

Upon diagnosis of (1) Neonatal Pneumonia, (2) Preterm Infant, and (3) Osteogenesis Imperfecta (Sillence Type III), a multidisciplinary management protocol was activated:

- Neonatology & Respiratory Therapy: Managed with nasal Continuous Positive Airway Pressure (nCPAP) (Figure 4). gradually weaned as the pneumonia resolved. The choice of non-invasive support was strategic, as endotracheal intubation carries heightened risks in OI patients, including potential cervical spine injury and difficult airway management.
- Nursing & Patient Safety: Implemented strict “handle with care” protocols, including prominent bed signs and minimized handling (4).
- Nutritional Support: Administered oral Vitamin AD, calcium gluconate, and mixed feeding, supplemented with parenteral nutrition to support metabolic demands and bone health (5). This coordinated effort led to successful weaning from respiratory support, full enteral feeding, and, most importantly, no new fractures during the 9-day hospitalization. The infant was discharged at a corrected gestational age of 36 weeks +5 days.

#### 2.4.2 Post-discharge phase: the first fracture and orthopedic follow-up

**Figure 5:**
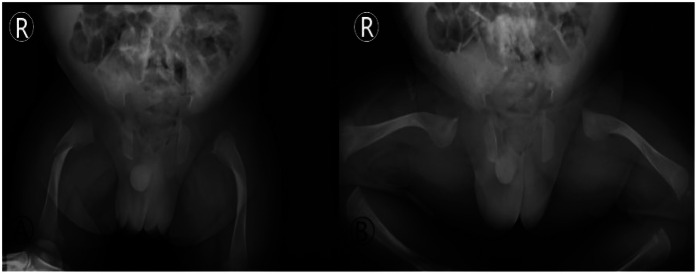
Imaging of the first spontaneous fracture post-discharge (day 25 of life). Anteroposterior (AP) and frog-leg lateral radiographs of the hips: **(A) (AP view)**: a fracture line with slight angulation is visible in the proximal right femur (red arrow). **(B) (FroG–Leg lateral view)**: the inherent bony abnormalities of OI, including bowing and thin cortices, are also seen. This image documents the first “traumatic” event during the home care phase, highlighting the high vulnerability during the post-discharge transition.

On the 16th day after discharge (25th day of life), the infant experienced a spontaneous fracture of the right proximal femur (Figure 5). The family promptly consulted our pediatric orthopedic team.

**Figure 6:**
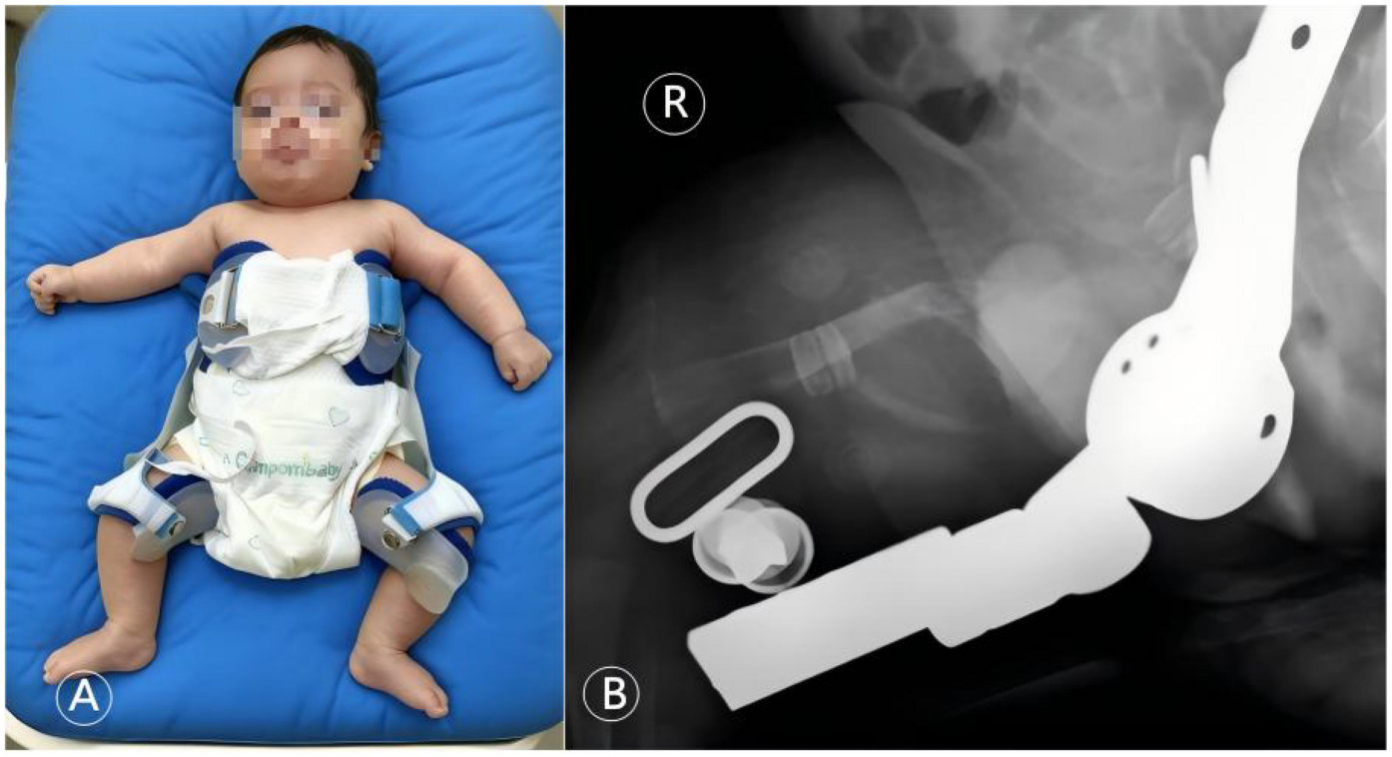
Orthopedic conservative management with a Pavlik harness. **(A)** Clinical photograph showing the infant with both lower limbs immobilized in a Pavlik harness. **(B)** Corresponding right femur radiograph taken with the harness in place, confirming maintained fracture alignment under fixation. This figure illustrates the key orthopedic intervention within the multidisciplinary team, employing a minimally invasive and effective stabilization method suitable for neonates with OI.

**Figure 7:**
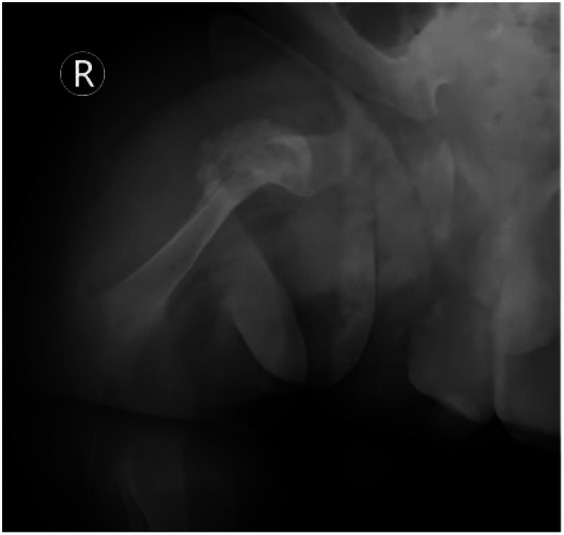
Radiographic evidence of rapid fracture healing (day 52 of life). Follow-up radiograph of the right femur reveals: satisfactory fracture alignment with a blurred fracture line and abundant cloud-like callus formation surrounding the site (red arrow). This image exemplifies the characteristic “rapid and hyperplastic” callus formation ability in OI, indicating successful initial healing and meeting the criteria for harness removal.

**Figure 8:**
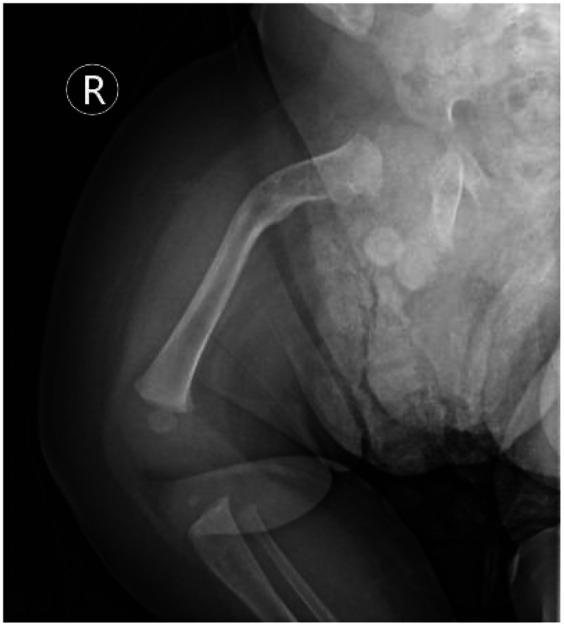
Radiographic outcome at the final follow-up (day 136 of life). Final follow-up radiograph of the right femur demonstrates complete bony union with remodeling and consolidation of the previously formed callus (red arrow). Despite fracture healing, the underlying bony deformity and bowing of the femur persist. This image shows the final outcome of the fracture and underscores the persistent nature of the skeletal abnormalities in OI, which coexist with the fracture healing process.

- Orthopedic Intervention: Conservative management with a Pavlik harness was initiated immediately to immobilize the limb in a safe, abducted position (Figure 6) (6).
- Healing Process: Follow-up radiographs at 52 days of age revealed abundant callus formation with blurred fracture lines (Figure 7), meeting the criteria for harness removal. By 136 days of age, radiographic union was confirmed, with completed callus remodeling, though the underlying bony abnormalities persisted (Figure 8). This rapid healing trajectory is characteristic of OI (7).

## 3 Discussion

### 3.1 Multidisciplinary management: bridging critical care and fracture prevention

Our case exemplifies the intricate balance required in managing a neonate with severe OI, where critical care for respiratory compromise must be delivered in the context of extreme skeletal fragility (1, 8). The successful in-hospital course, free of iatrogenic injury, underscores the effectiveness of a vigilant, protocol-driven MDT approach in the controlled NICU environment (4, 5).

### 3.2 Respiratory system involvement in OI: specific considerations and management strategies

Managing the respiratory system in neonates with osteogenesis imperfecta (OI) necessitates specialized approaches that diverge from standard neonatal protocols. Our patient exhibited respiratory distress, weak breathing effort, and a short thorax, with oxygen saturation falling to 85% by the ninth day after birth. Due to the substantial risks invasive procedures pose for OI patients—such as cervical spine injury, odonto-axial dislocation, and fractures of the mandible or teeth during laryngoscopy—we opted against immediate endotracheal intubation. Instead, a carefully titrated, non-invasive strategy was employed. Following a deterioration in his condition and computed tomography (CT) evidence of worsening pneumonia, he was commenced on nasal Continuous Positive Airway Pressure (nCPAP) with the following settings: PEEP 5 cmH_2_O, FiO_2_ 30%. This intervention successfully stabilized his respiration, thereby avoiding the need for invasive mechanical ventilation. This outcome aligns with literature suggesting noninvasive positive pressure ventilation (NPPV) can manage acute respiratory failure in OI while circumventing intubation-associated complications (9). As he improved, respiratory support was systematically reduced to high-flow nasal cannula (HFNC at 4 L/min, FiO_2_ 25%) and was ultimately discontinued on day 18 of life. The pathophysiology of respiratory compromise in these infants involves a combination of intrinsic pulmonary issues from defective type I collagen in the lung matrix and extrinsic restrictive forces caused by thoracic skeletal deformities, such as the short thorax and altered spinal curvature noted in our case (10). The efficacy of this staged, non-invasive protocol highlights the importance of customizing respiratory support for neonatal OI to reduce the considerable dangers posed by invasive airway procedures in this fragile group.

### 3.3 Genetic and orthopedic trajectory in severe neonatal OI

The COL1A2 c.1459G > A (p.Gly487Arg) mutation in our patient is a typical glycine substitution within the triple helix, leading to a structural defect in type I collagen, which explains the severe phenotype (Sillence III) observed (1, 11). The spontaneous fracture that occurred shortly after discharge starkly highlights the transition from the protected hospital setting to the home environment as a period of significant risk (12). This transition represents a critical gap that our current healthcare systems must address more robustly. It mandates not only comprehensive family education on safe handling techniques, recognition of fracture signs, and emergency response but also a seamless handover to community and outpatient services to ensure continuity of care (4). The fracture healing observed in our patient—rapid callus formation followed by remodeling—is a hallmark of OI and has been documented in the perinatal period (7, 13). While this robust healing response is beneficial for fracture union, it can also lead to malalignment if not properly managed. Conservative management with devices like the Pavlik harness is often the mainstay for diaphyseal fractures in infants with OI, providing adequate stability while allowing for this unique biological process (6, 10).

### 3.4 The critical transition: post-discharge vulnerability and caregiver perspectives

The spontaneous fracture that occurred 16 days after discharge offers a critical insight from our case. This complication highlights the immediate post-discharge phase as a time of significant vulnerability, pointing to a substantial deficiency in existing care continuum models. Our findings indicate that thorough family education—which must incorporate practical training in safe handling, accessible emergency contacts, and explicit instructions for identifying signs of a fracture—forms the foundation for a secure transition home. This component is a cornerstone of patient safety, equivalent in importance to the management provided within the hospital. The parents involved described considerable anxiety, especially concerning techniques for swaddling, carrying, and changing diapers safely. Their experience stresses the necessity of enhanced, direct instruction and reinforcement of these skills prior to discharge.

### 3.5 Strengths, limitations, and comparative context

The principal strengths of this report encompass thorough genetic confirmation, extensive longitudinal imaging that tracks disease progression and fracture healing, and clear reporting on the implementation of a Multidisciplinary Team (MDT) approach from the NICU through the high-risk post-discharge phase. Nevertheless, as a single-case study, the findings possess inherent limitations regarding their generalizability. In comparison to other published accounts of neonatal osteogenesis imperfecta (OI) (2, 4, 12), our work delivers distinctive perspectives on the management of an infant with a confirmed COL1A2 mutation. It particularly stresses the necessity of prolonging the MDT model of care after the patient leaves the hospital. Although the specific interventions described may be established practice, the systematic account of this integrated care pathway and its explicit focus on the risks following discharge offer clinically actionable insights for teams faced with analogous situations.

### 3.6 Future directions in OI management

Looking forward, the long-term management of children with severe OI requires a sustained MDT effort to address progressive skeletal deformities, potential pulmonary complications, and functional limitations (14, 15). While bisphosphonate therapy is often considered after the neonatal period, emerging therapies like mesenchymal stem cell transplantation represent promising future avenues (16). Our experience reinforces that future clinical protocols should specifically address the high-risk post-discharge period through standardized transition pathways, enhanced caregiver training, and closer early follow-up arrangements.

## 4 Conclusion

In conclusion, this case report illustrates that the management of severe neonatal OI is a continuous journey that begins in the NICU and extends far beyond discharge. A proactive, multidisciplinary strategy is paramount for initial stabilization and in-hospital fracture prevention. However, our experience confirms that the post-discharge period is a vulnerable window for traumatic fractures. Therefore, building a robust support system that includes thorough family education and seamless follow-up care is essential to safeguard these vulnerable infants and optimize their long-term functional outcomes.

## Data Availability

The datasets presented in this study can be found in online repositories. The names of the repository/repositories and accession number(s) can be found in the article/Supplementary Material.
